# Multi-Kernel Temporal and Spatial Convolution for EEG-Based Emotion Classification

**DOI:** 10.3390/s22218250

**Published:** 2022-10-27

**Authors:** Taweesak Emsawas, Takashi Morita, Tsukasa Kimura, Ken-ichi Fukui, Masayuki Numao

**Affiliations:** 1 Graduate School of Information Science and Technology, Osaka University, Osaka 565-0871, Japan; 2 The Institute of Scientific and Industrial Research (ISIR), Osaka University, Osaka 567-0047, Japan

**Keywords:** brain–computer interface (BCI), electroencephalography (EEG), emotion classification, machine learning, convolutional neural network (ConvNet)

## Abstract

Deep learning using an end-to-end convolutional neural network (ConvNet) has been applied to several electroencephalography (EEG)-based brain–computer interface tasks to extract feature maps and classify the target output. However, the EEG analysis remains challenging since it requires consideration of various architectural design components that influence the representational ability of extracted features. This study proposes an EEG-based emotion classification model called the multi-kernel temporal and spatial convolution network (MultiT-S ConvNet). The multi-scale kernel is used in the model to learn various time resolutions, and separable convolutions are applied to find related spatial patterns. In addition, we enhanced both the temporal and spatial filters with a lightweight gating mechanism. To validate the performance and classification accuracy of MultiT-S ConvNet, we conduct subject-dependent and subject-independent experiments on EEG-based emotion datasets: DEAP and SEED. Compared with existing methods, MultiT-S ConvNet outperforms with higher accuracy results and a few trainable parameters. Moreover, the proposed multi-scale module in temporal filtering enables extracting a wide range of EEG representations, covering short- to long-wavelength components. This module could be further implemented in any model of EEG-based convolution networks, and its ability potentially improves the model’s learning capacity.

## 1. Introduction

### 1.1. Background

The brain—computer interface (BCI) technology is a technology that acquires brain signals and interprets neuronal information into the desired action. BCI has been used in various medical and non-medical applications [1], such as assistive technology [2,3], game playing [4,5], and mental state recognition [6,7]. There are several ways to record brain activity. One of the most popular modalities of BCI is electroencephalography (EEG). The EEG method has been used to record electrical signals in the human brain by measuring tiny voltage fluctuations using electrode sensors. The EEG recording can be performed by attaching electrodes to the scalp surface without surgery and implantation. This non-invasive EEG system holds the promise of real-world BCI applications and is currently entering the mass market. For example, EEG has been used to diagnose abnormalities of the human brain by detecting the presence of aberrant electrical activity [8,9]. In non-medical fields, attempts have been made to transduce EEG states from human players to control video games [10]. Moreover, the acquisition of EEG signals is also exploited to recognize human psychological states [11]. Distinct patterns of EEG signals can describe users’ emotions and feelings in response to specific circumstances with less human bias. Despite its several potential applications, the non-stationarity and high-dimensionality of EEG signals pose a challenge to reliable implementation. EEG recordings could be easily affected by various sources of noise, including eye movement, muscle contraction, and environmental settings, which present significant difficulties in EEG interpretation. Because the brain is a complex organ with different parts that function and respond differently, to evaluate spatial brain activity, EEG data are generally recorded in more than one and up to 64 electrode positions increasing space dimensionality and complexity during feature extraction and analysis.

Therefore, the capability of developed EEG methods should consider several aspects, including interpretability, performance, and usability for real-world applications. Ascertaining methods aim to understand complex EEG signals derived from the human brain extracting their informative features and classifying them. Conventionally, beneficial knowledge is extracted from raw EEG signals by manually defined features and reported through statistical reports. For instance, frequency bands in slow to hyperactivity brain waves reflect the distinction in the intensity of emotions [12]. Positive emotions, such as joy or happiness, are relatively associated with the left frontal hemisphere. In contrast, negative emotions, such as sadness or fear, are relatively associated with the right frontal hemisphere [13,14]. Nevertheless, these defined features are easily sensitive to noise, and their computation requires appropriate data cleansing, signal preprocessing, and expertise.

### 1.2. Research Gap

Recently, many studies have applied deep learning (DL) to extract and interpret EEG signals. An end-to-end convolutional neural network (ConvNet or CNN) is constructed on the basis of a shared-weight architecture that can reasonably learn joint optimization of feature extraction and classification. The appropriate design, such as the layer’s depth and width, kernel size, and optimizer, becomes an essential consideration that significantly affects the representational ability and classification performance. It has been demonstrated that deep and shallow convolution networks can automatically extract essential temporal and spatial information from raw EEG signals [15]. Nevertheless, as the feature extraction of these models is based on a single kernel size, their learning ability and EEG representational performance are limited. Larger ranges of signal transformations are needed to represent differences in slow and hyperactive EEG frequencies. Parameter tuning and optimization are also required for a particular task. In addition to the kernel, parameter dependence and computational cost in training and testing processes have to be taken into account [16]. A separation of network layers was suggested to help reduce the computational cost and model complexity. To further improve model efficiencies, the network architectures, including kernel sizes and parameter sets, should be rationally designed for each specific EEG analysis task.

### 1.3. Motivation and Contribution

Accordingly, achieving good EEG data analysis performance using DL-based techniques requires a wide range of useful representations while preserving optimized trainable parameters. This study proposes an architecture called multi-kernel temporal and spatial convolution for EEG-based emotion classification. We present (1) multi-kernel learning for temporal convolution and (2) filter recalibration with a lightweight gating mechanism. The proposed model makes the classification process more efficient by factoring the kernel into a series of operations to capture various short and long patterns. Then, separable convolution is applied for spatial learning by considering each channel separately and convolving over electrode channels. Moreover, we recalibrate weights after temporal and spatial convolving to utilize the limited data available. We compare our model with existing techniques and investigate task accuracy by conducting classification experiments on BCI datasets.

## 2. Related Works

### 2.1. Feature Extraction and Classification

**Figure 1:**
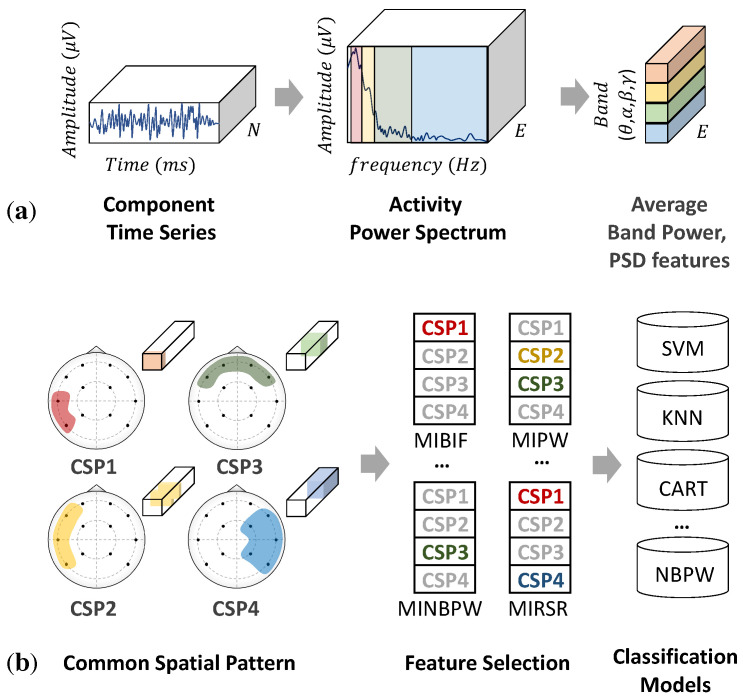
(**a**) Power spectral density (PSD) features, extracted from raw EEG signals. (**b**) Filter bank common spatial pattern (FBCSP).

In the classification of emotions learned from EEG signals, feature extraction is an essential step in which these emotions are represented and categorized into the desired labels. Conventionally, EEG features can be categorized into three domains: time, frequency, and time–frequency domains [17]. The time domain observes time-series characteristics and variations using conventional techniques, such as linear prediction and component analysis. The frequency or spectral domain is the standard method used for quantifying EEG signals by adapting the Fourier transform. Fourier analysis reflects the frequency content using sums of trigonometric functions and then distributes the average power into the PSD. As shown in Figure 1a, the PSD in a human EEG signal is approximately divided into several ranges, including theta (4–7 Hz), alpha (8–12 Hz), beta (13–32 Hz), and gamma (>32 Hz) bands. These frequency bands reflect brain activities through the strength of variation. High-frequency bands, such as gamma or beta waves, indicate hyperactive brain activity and alertness, and low-frequency bands, such as delta or theta waves, indicate deep meditation and relaxation. Theta (4–7 Hz) is a slow wave associated with the subconscious mind, deep relaxation, and meditation. Changes in alpha (8–12 Hz) and beta (13–32 Hz) waves are the most discriminative for emotional states [12,18]. Gamma (>33 Hz) is a hyperactivity wave associated with problem-solving and concentration and is related to positive and negative emotions but on different sides; left for negative and right for positive [19].

Moreover, in addition to frequency or temporal features, spatial features can be extracted using multi-channel electrodes. Each part of the human brain can convey different information through the asymmetric hemisphere. For example, the frontal lobe is associated with reasoning, parts of speech, and emotion. The temporal lobe is associated with the perception and recognition of auditory stimuli, whereas the occipital lobe is responsible for vision. For this reason, EEG signals require a method that can manipulate both temporal and spatial information for feature learning and classification. A common spatial pattern (CSP) [20] is a mathematical method for computing the variance of features to discriminate window patterns or emotional classes. This method uses the simultaneous diagonalization of two covariance matrices to construct optimal spatial filters [21,22]. CSP patterns can be used as features for machine learning (ML) to classify emotions [23]. However, the classification of CSP features requires a specific frequency range, which significantly depends on the subject or task. To address this problem, filter bank common spatial pattern (FBCSP) [21] has been proposed to perform an autonomous feature selection through temporal-spatial filtering. Compared with CSP methods, FBCSP as shown in Figure 1b, adds two more processes to perform feature selection and classification, respectively. The first two stages perform temporal and spatial filtering to construct a filter bank of discriminative CSP features. Then, features are selected independently depending on the classifiers in the third stage. Popular techniques with good EEG classification accuracy include random forest (RF), K-nearest neighbor (KNN), support vector machine (SVM), and fully connected network (FCN) [24,25]. However, these techniques employ manual parameterization to classify EEG features, and the appropriate selection for a particular task relies on the complexity and cleanliness of data recording.

### 2.2. End-to-End Convolutional Neural Networks

**Figure 2:**
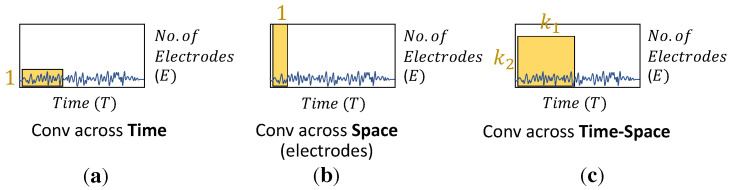
EEG convolution filter types include (**a**) temporal filter, (**b**) spatial filter, and (**c**) temporal—spatial filter. The yellow blocks denote the two-dimensional (2D) kernel for performing feature extraction.

In recent years, the use of end-to-end CNNs (ConvNets) has been introduced for effective and reasonable EEG analysis in feature extraction and classification tasks. First, the raw EEG signals are measured in microvolts (μV) and recorded in a 2D space, with temporal (*T*, time) and spatial dimensions (*E*, the number of electrode channels). Then, the convolutional operation is applied to extract informative features through three types of filtering, i.e., convolving across time, space, and both time and space, respectively, as shown in Figure 2. Temporal filtering convolves information across the time—space domain with a 1×t kernel size. The temporal content of each electrode channel is extracted by shared weighting over the time—space input. Spatial filtering proceeds with the filter matrix E×1 across all electrode channels, learning the variance of features. The temporal—spatial kernel with a $k_{1}\times k_{2}$ 2D kernel size, where *k* represents a specific kernel size, is applied to both time and channel domains. In addition, there is a need to determine the suitable size for learning various representations and distinctions. Typically, the kernel size is manually defined and used in feature extractors in CNNs. A single-scale kernel might be trapped with a limited amount of representations. Especially for EEG feature extraction, the learning of representations needs to be diverse and capable of effectively extracting temporal and spatial features.

**Figure 3:**
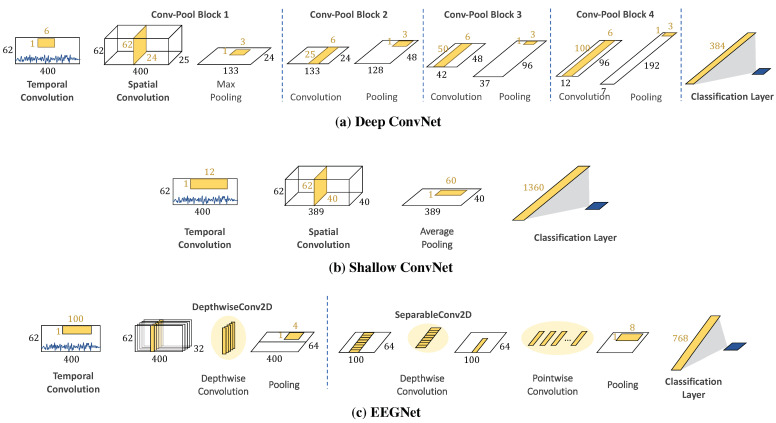
Model architectures of (**a**–**c**).

Apart from kernel designs, the network depth significantly influences the learning strategy, particularly low–high-level feature decoding. For example, a deep convolutional network (Deep ConvNet) has been used for EEG decoding with a single-layer temporal filter, and then, the output is fed into multi-layer spatial convolution and pooling layers, as shown in Figure 3a. The EEG classification results show that the Deep ConvNet outperforms the widely used FBCSP (mean decoding accuracy of 84.0% and 82.1%, respectively) [15]. Deep ConvNets can achieve competitive accuracy and can be applied to general EEG decoding tasks. On the other hand, according to the FBCSP pipeline, a shallow convolutional network (Shallow ConvNet) is designed for tailoring decoding band power features, as shown in Figure 3b. The first layer performs a temporal convolution to simulate bandpass extraction. Then, the second layer performs a spatial convolution that analogizes the CSP spatial filter in FBCSP. The extracted features of Shallow ConvNet are related to log band power and are designed explicitly for oscillatory signal classification.

Moreover, another critical aspect that affects informative features and learning ability is reducing the network size while maintaining good performance. One of the simpler networks is a separable convolution that enables networks to construct informative features by fusing both spatial and channel information. The operation consists of spatial mapping independently performed over each input channel and feed-forward mapping to project the output onto a new feature space. These operations enable networks to construct informative features by fusing spatial and temporal information within local receptive fields at each layer. A compact CNN for EEG-based BCI, called EEGNet, is introduced to construct an EEG-specific model with separable convolution [16]. The number of trainable parameters in EEGNet is significantly less than that of Deep ConvNet and Shallow ConvNet (170 and 100 times, respectively), but EEGNet still achieves performance comparable to that of Deep ConvNet and Shallow ConvNet.

## 3. Proposed Method

**Figure 4:**
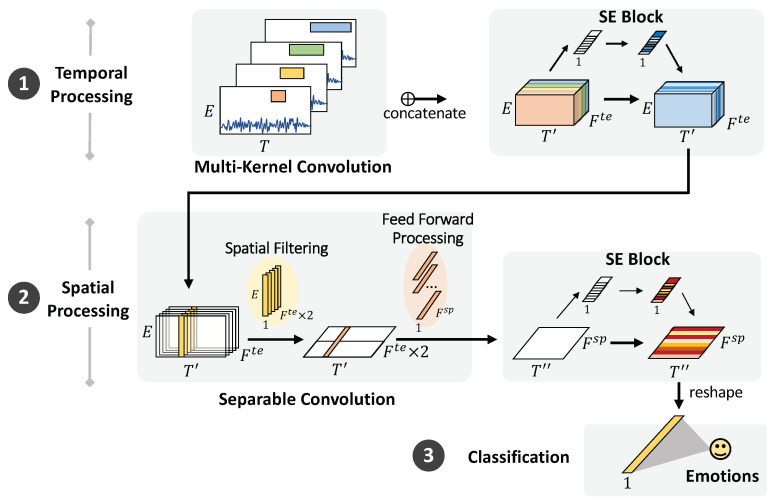
MultiT-S ConvNet architecture. The model comprises temporal filtering, spatial filtering, and classification layers. The first layer employs four types of temporal convolutions to learn multiple temporal features from raw EEG signals. Then, the separable convolution is used to learn temporal-specific spatial filters across electrode channels. The SE block is used to recalibrate the features of both filters. The final classification layer is used to discriminate the emotional labels.

This study proposes an end-to-end CNN called multi-kernel temporal and spatial convolution (MultiT-S ConvNet) for EEG-based emotion recognition. The proposed model enables multi-scale representation learning and improves classification performance. The model consists of three parts, namely, a temporal learner, spatial learner, and classifier, which simultaneously learn discriminative representations in the time and electrode channel dimensions. The model architecture is depicted in Figure 4, and the details are described in the following sections.

### 3.1. Multi-Kernel Temporal Processing

In this study, we hypothesize that multi-kernel filters can improve temporal representations learned from raw EEG data. Considering the kernel design of temporal convolution, the larger size can learn higher resolutions in the time domain but necessitates high computational costs. On the other hand, a smaller kernel size essentially learns low-level features or shorter temporal patterns in the time domain and can reduce computational costs. Multi-scale convolution kernels combining long and short patterns are applied by factoring a kernel into various kernel sizes that would independently convolve and map them in order. Learning a wide range of representations while avoiding high computational costs can improve the performance of EEG classification. The main advantage of this architecture is significant quality gain at a modest increase in computational requirements compared with shallower and narrower architectures. Accordingly, we specifically adopted four kernels of length 25 ms (=5 samples under the sampling rate of 200 Hz), 50 ms (=10 samples), 100 ms (=20 samples), and 200 ms (=40 samples). These choices were based on four frequency bands, namely, theta (4–7 Hz), alpha (8–12 Hz), beta (13–32 Hz), and gamma (>33 Hz), extensively used to characterize brain states/activities. The sampling rate is also considered because wavelengths can be captured with the same time scale, even from different datasets. These multi-kernels can capture an extensive range of representations in EEG signals. The larger kernel size can learn various informative features, along longer temporal patterns. Meanwhile, the smaller kernel size specifically extracts shorter temporal patterns.

The raw EEG signals in Figure 4 can be represented as 2D time series whose dimensions are time (T) and electrode channels (E). The window size is set as 2 s (*T* is 400 data points for the SEED dataset) based on the change in emotion over time. We duplicate the input into four modules and convolve them using four different kernel sizes: (1×5), (1×10), (1×20), and (1×40). Each module is padded with zeros to retain the same size. Along the multi-kernel temporal convolution, a tensor of size $\left(T^{\prime }\times E\times F^{te}\right)$ is produced, where $T^{\prime }\times$ denotes the temporal dimensionality after convolution and $F^{te}$ denotes the total number of temporal features after concatenation. All convolutions, including those modules, apply rectified linear activation (ReLU) and average pooling to data before sending them to the next layer.

### 3.2. Remaining Modules

#### 3.2.1. Spatial Processing

**Figure 5:**
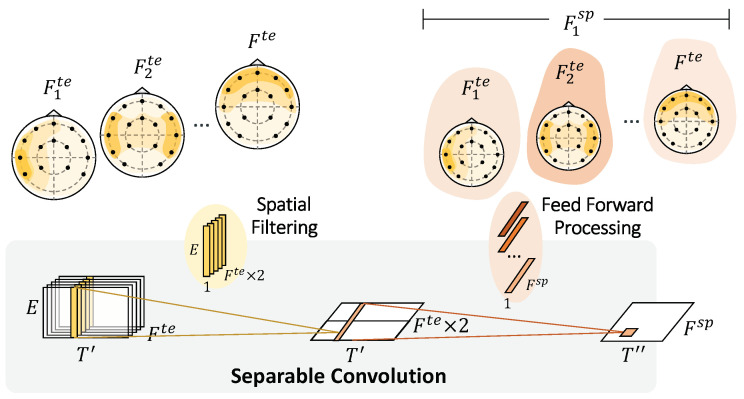
Spatial Convolution. Spatial processing separately convolves across the electrodes on the temporal feature maps. Feedforward processing subsequently weights the feature maps to extract temporal—spatial features. Shading represents how the network emphasizes or understates the corresponding feature maps.

For each time step ($T^{\prime }$) and temporal feature ($F^{te}$), we collected information across electrodes using two full-length filters. A separable convolution was applied for spatial feature learning and to reduce computational costs. Figure 5 depicts the separable convolution and the virtualization of topoplots. It consists of two steps: spatial filtering and feed-forward processing. Spatial filtering connects each temporal feature map individually to learn specific spatial filters across electrodes. We manually set the feature multiplier to 2 to increase the number of parameters in the neural network to learn more traits better. The network gathers data with the $F^{te}\times 2$ of the respective filter (1×E) and stacks the outputs into feature maps $\left(T^{\prime }\times 1\times \left(F^{te}\times 2\right)\right)$. The interpretation of this step is to build multiple filters that can learn informative features across all electrode channels individually. Then, feed-forward processing continues to learn and optimizes a temporal—spatial summary for each feature map with independent filters $\left(1\times 1\times \left(F^{te}\times 2\right)\right)$ across the spatial feature maps. Here we learn how to weigh the useful set of filters from the previous extraction. After this spatial processing, temporal—spatial feature maps $\left(T^{\prime \prime }\times 1\times F^{sp}\right)$ are extracted, where $T^{\prime \prime }$ represents the time after temporal—spatial processing and pooling.

#### 3.2.2. Channel Recalibration

From the outputs, after each temporal and spatial processing, we need to utilize the limited dataset as much as possible regarding the time-cost consumption of EEG recording. The squeeze-and-excitation block (SE block) is further applied to adaptively recalibrate the gathering of informative feature responses by explicitly modeling interdependencies between feature maps [26]. Adding SE blocks after the convolutional layers can help model weighting that learns to emphasize and dismiss informative features. The weights of each feature map are equally informative after performing temporal convolution in Section 3.1. The temporal SE block is briefly described in two steps. First, in the squeezing step, global information is compressed into a feature descriptor $\left(1\times 1\times F^{te}\right)$ using average pooling. Next, a dense layer is added with ReLU activation to reduce the channel complexity by a ratio (r) while *r* is set to 16. Then, another dense layer with sigmoid activation is added to give each channel a smooth gating function. Finally, the excitation step obtains the aggregated information and fully captures the channel-wise dependencies. According to the temporal feature maps, the features are multiplied by the weights of the temporal SE block and output $\left(T^{\prime }\times E\times F^{te}\right)$.

Moreover, after spatial processing in Section 3.2.1, the spatial SE is applied to adaptively recalibrate temporal-spatial feature responses. The squeeze step is to compress global temporal-spatial information into a feature descriptor $\left(1\times 1\times F^{sp}\right)$. Next, two dense layers reduce the channel complexity (r=16). In the squeezing step, the temporal-spatial feature maps are multiplied by the weights of the spatial SE block. After performing these convolutions, the extracted features are generated and transmitted to the classification layer.

#### 3.2.3. Classification

The outputs from the previous module are flattened into one vector representing all extracted features and linearly transformed into classification logits with an FCN. For binary classification, the final layer unit is 1. The loss function is binary cross-entropy, and the activation function is a sigmoid function. While 3 classes classification, the final layer unit is 3. The loss function is categorical cross-entropy, and the activation function is a softmax function. The final output is a discrete emotion label with probability.

## 4. Experiments and Results

To validate the method’s performance, feature extractability, and classification accuracy, we examined and compared it with existing methods. The experiments and results consist of subject-dependent and independent classification on the SEED and DEAP datasets.

### 4.1. Experiment Setting

#### 4.1.1. Datasets

This study conducted experiments on two open-access datasets: SEED (SJTU emotion EEG dataset) [22] and DEAP (dataset for emotion analysis using physiological signals) [27] datasets. These datasets have been widely used in emotion recognition using multimodal physiological signals. The SEED dataset [22] consists of 64 channels of EEG signals recorded from 15 subjects. All subjects were asked to watch 15 excerpts of movie clips for 3 trials. Each clip was approximately 4 min long, and the time interval between trials was one week or longer. The emotional labels corresponded to three types of movies to stimulate emotional states: positive, neutral, and negative. The recorded EEG signals were downsampled from 1000 to 200 Hz and applied a bandpass filter from 0–75 Hz. The DEAP dataset [27] is a multimodal dataset that consists of EEG and peripheral physiological signals. The EEG signals of 32 subjects were recorded by 32 electrodes using the BioSemi ActiveTwo system; 40 one-min excerpts of music videos were used to stimulate emotional states. All subjects were asked to rate the emotion score (1–9) in the valence-arousal space. The EEG signal was recorded with a 512 sample rate. The signals were downsampled to 128 Hz, a bandpass filter was applied to them from 4 to 45 Hz, and EOG artifacts were removed. According to the review paper [25], the average accuracy of the SEED dataset was 90.0%, significantly higher than 83.6% of the DEAP dataset within-subject dependencies.

This study conducted SEED experiments in a three-class classification, including positive, neutral, and negative labels, and a binary classification with less complexity and fewer data considered positive and negative labels. On the other hand, DEAP employed 2D models rated by subjects caused discrepancies between movie types and real subject emotions. To simplify the problem, we investigate a binary classification of valence and arousal scores with positive and negative labels.

#### 4.1.2. Baseline and Existing Models

In baseline experiments, the PSD with four bands was used as extracted features, along with ML classifiers. The baseline classifiers related to EEG analysis comprise the KNN, RF, and FCN. These models were tuned by grid search with the optimal hyperparameters selected, resulting in the most accurate performance. Accordingly, the number of neighbors for the KNN classification is set to 21. The number of RF trees is set to 100. The FCN has 2 hidden layers with 100 nodes, which are then forwarded to the output layer. The activation function is the logistic sigmoid function. Adam optimization is applied while training the FCN model.

**Figure 6:**
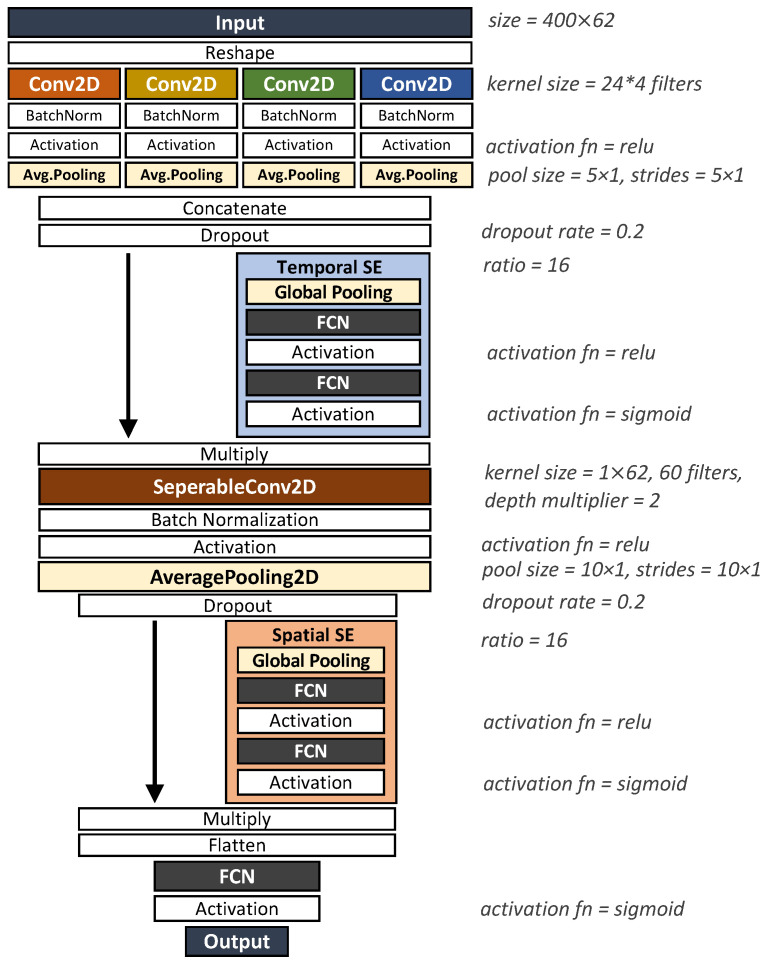
MultiT-S ConvNet model setting.

Moreover, we compare the performance of MultiT-S ConvNet against existing models, including the Deep ConvNet, Shallow ConvNet, and EEGNet models. The Deep ConvNet architecture [15] consists of one temporal convolution layer, four spatial convolution and pooling layers, and a classification layer in order. The Shallow ConvNet architecture [15] consists of single temporal and spatial convolution layers. Then, it sequentially passes data to a pooling layer and a classification layer. For the EEGNet architecture, we refer to the original network [16], which consists of temporal convolution and a separable convolution layer followed by a classification layer. The MultiT-S ConvNet architecture is depicted in Figure 6. The number of filters and trainable parameters are shown in Figure 2. We applied the Adam optimizer to all DL models in the training process. The loss function for binary classification is binary cross-entropy, whereas three-class classification is categorical cross-entropy. Each model was trained for 200 epochs with 100 batch sizes. These were trained on GPU, NVIDIA Quadro P6000 (Santa Clara, United States), using Tensorflow [28] and Keras [29].

#### 4.1.3. Comparison Approaches

All models were examined on the subject-dependent and independent classification of the SEED and DEAP datasets. In the subject-dependent experiments, 5-fold cross-validation was applied, experimenting with different partitions of 80% training data and 20% test data; 30% of the training data were held out for validation of free parameters, and the model parameters were optimized on the basis of the remaining 70%. Moreover, the validation set was randomly picked on the desired subject, and the 5-fold cross-validation was applied to all subjects. For the subject-independent experiment, we applied the leave-one-out cross-validation to access model performance. The model was trained using data from all but one subject and tested on the held-out subject; 30% of the training data were randomly selected for free-parameter validation without balancing among subjects.

The performance metric was classification accuracy. Moreover, we show the chance level, which is the obtained accuracy when consistently predicting the majority class. To verify multiple comparisons, analysis of variance (ANOVA) was applied to determine whether the means in the desired group were significantly different. Then, Dunnett’s test was used to observe the many-to-one comparisons with our proposed method.

### 4.2. SEED Experiments and Results

**Figure 7:**
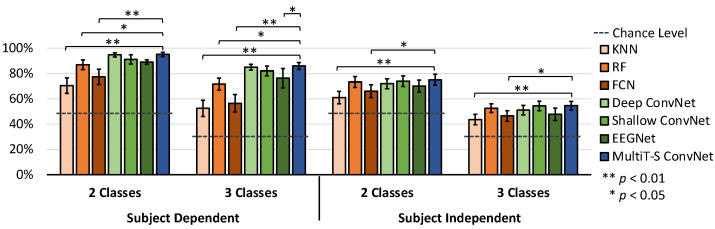
Average classification accuracy for all subjects on the SEED dataset. Error bars denote a 95% confidence interval (CI) computed from all subject’s means. The horizontal dashed lines indicate the chance level. The stars indicate significant differences compared with MultiT-S ConvNet.

For the SEED dataset experiments, we performed two-class and three-class classification to study both subject-dependent and subject-independent results. The two-class experiment uses two-thirds of the three-class dataset to simplify the problem’s complexity and additional investigation. Their accuracy is shown in Figure 7 and Table 1. For the performance results, all models can achieve the 50% and 33.3% chance levels in the subject-dependent and subject-independent experiments, respectively. We used one-way ANOVA to compare differences in the means of all models, and we found that the SEED dataset’s results are significant. The post hoc Dunnett’s test is then conducted to compare every result with that of MultiT-S ConvNet for observing the significant difference. RF, Deep ConvNet, Shallow ConvNet, EEGNet, and MultiT-S ConvNet significantly outperform the chance levels (*p* < 0.01). Moreover, MultiT-S ConvNet achieves the highest accuracy in all experiments. For two-class and three-class classifications, the subject-dependent results are 95.2 ± 3.2% and 86.0 ± 5.3% respectively, whereas the subject-independent results are 75.1 ± 8.4% and 54.6 ± 6.8% respectively. RF outperforms other baseline techniques by a significant level (*p* < 0.05). The accuracy of all DL models is significantly higher than KNN (*p* < 0.01) and FCN (*p* < 0.05) models. However, there is no statistical difference among deep learning models (*p* > 0.05), except in three-class and subject-independent experiments, where the accuracy of MultiT-S ConvNet is significantly higher than that of EEGNet (*p* < 0.05).

### 4.3. DEAP Experiments and Results

**Figure 8:**
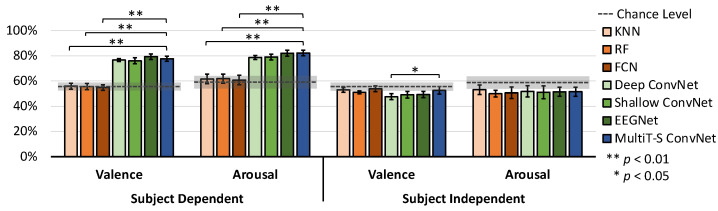
Average classification accuracy for all subjects on the DEAP dataset. Error bars denote a 95% CI computed from all subject’s means. The horizontal dashed lines indicate the chance level, and the grey areas indicate the CI of chance levels. The stars indicate significant differences compared with MultiT-S ConvNet.

Using the DEAP dataset, we conducted four experiments of valence and arousal binary classification on both subject dependence and subject independence. The classes were defined by emotion scores, where 1 to 4 is a negative class, and 5 to 9 is a positive class. DEAP dataset’s results are shown in Figure 8 and Table 1. Similar to the SEED dataset, one-way ANOVA with Dunnett’s post hoc is applied to investigate significant differences among all models. The chance level of valence is 56.6 ± 9.2%, whereas the chance level of arousal is 58.9 ± 15.5%. For subject-dependent experiments, EEGNet outperforms the other models with a valence classification accuracy of 79.2 ± 5.8%, and MultiT-S ConvNet outperforms the other models with an arousal classification accuracy of 82.2 ± 6.5%. In both valence and arousal experiments, all CNN models explicitly and significantly outperform the baseline models (*p* < 0.01). Nevertheless, these chance levels could not be achieved in the subject-independent experiments.

### 4.4. Ablation Study

To verify the effectiveness of our temporal filtering, we further investigate the effect of temporal convolution on the other CNN models. Based on the first blocks of each model in Figure 3, we reform a temporal convolution with multi-kernel convolution. Meanwhile, the spatial filters were halved to preserve the number of parameters and computational cost. Here, we consider a two-class classification with a subject-independent setting on the SEED dataset, and the results are shown in Table 2. In addition, a paired t-test is used to observe the difference between the corresponding models. Deep ConvNet with multi-kernel temporal convolution consists of 12 × 4 temporal (band) filters, 12 spatial filters, and 168 temporal-spatial filters. As a result, the number of trainable parameters decreases from 182,497 to 73,777. It significantly outperforms the original Deep ConvNet with 73.96% accuracy from 72.04% (*p* < 0.05). For EEGNet with multi-kernel temporal convolution, the number of trainable parameters increases from 13,537 to 16,177, but it achieves a significantly better accuracy of 74.00% than the original EEGNet of 70.15% (*p* < 0.01). However, there is no significant difference between Shallow ConvNet with and without multi-kernel convolution (*p* > 0.05). Moreover, we trained the MultiT-S ConvNet model without SE blocks to observe their effects on the learning performance. The accuracy of our model without SE Blocks decreases from 75.13% to 73.95% (*p* < 0.05).

## 5. Discussion

### 5.1. Classification Performance

In this study, the accuracy performance was examined using SEED and DEAP, which are well-known EEG emotion datasets. In addition, existing models and our proposed model were implemented with similar settings, including data segmentation, pre-processing, parameter tuning, and evaluation metrics, to ensure the reliability of model performance comparison. The experiments and results demonstrate that all ConvNets with appropriate design choices can outperform traditional approaches in terms of accuracy performances in all classification experiments. Moreover, this study compares MultiT-S ConvNet against existing ConvNet models in terms of accuracy performance in EEG-based emotion classification, as shown in Table 3. The comparison shows that the overall performance of MultiT-S ConvNet outperforms Deep ConvNet, Shallow ConvNet, and EEGNet with higher accuracies of 2.2%, 2.3%, and 3.7%, respectively.

According to the result, the SEED dataset is more uncomplicated to classify than the DEAP dataset because it contains different sources of emotional labels and the cleanliness of the signals. For the emotion states, the SEED dataset uses the movie types, whereas the DEAP dataset uses a subject questionnaire. This ambiguity directly affects the classification performance and trustworthiness of each dataset. Based on the SEED dataset results, all the obtained accuracy reached the chance level (*p* < 0.01) in both subject-dependent and independent experiments. For the DEAP dataset, only the ConvNet-based techniques reached the chance level (*p* < 0.01) in the subject-dependent experiments. In contrast, for the DEAP dataset, none of the models could surpass the chance level in subject-independent experiments. Many studies using the DEAP dataset have mainly conducted subject-dependent experiments because the data distribution of each subject in this dataset is highly diverse and varied. Observing the topoplots of PSD features in 4 frequency bands by human eyesight shows that the SEED dataset plots can be distinguished, whereas the DEAP dataset plots are very similar. In terms of subject-dependent and subject-independent learning, subject-dependent performance outperforms subject-independent performance significantly in all experiments. Consequently, the individual distribution directly affects learning performance. Moreover, our results are comparable and consistent with existing studies in terms of average accuracy [25].

### 5.2. Architectures and Design Choices

For several decades, ML has been applied in EEG analysis for individual modules combined with prior knowledge. Most previous studies applied manual parameterization for feature extraction and then transmitted them to an ML classifier [9,11]. These traditional techniques require background knowledge for signal processing, noise reduction, and data manipulation. From the results in terms of the PSD feature, the classifiers are possibly influenced and affected by the noise and complexity of multi-channel EEG signals, resulting in poor performance. However, the ensemble model, RF, performs well overall and significantly outperforms all baseline techniques on the SEED dataset. The RF model potentially learns how to reduce the generalization error of the prediction and deal with various EEG classification tasks.

On the other hand, ConvNet models are a better choice in EEG analysis than manual hand-crafted features. Numerous ConvNets with end-to-end architecture have been proposed to learn informative features and deal with the variation of noises in EEG analysis automatically and efficiently. For instance, attention classification using Shallow ConvNet and long short-term memory (LSTM) network on a three-back task [30], and emotion classification using subject-invariant bilateral variational domain adversarial neural network [31]. However, EEG features need to be extracted into various representations that improve the learning effect, especially for EEG analysis. Here, we proposed the multi-kernel convolution that helps the model achieve better results than the single-kernel convolution. As a result, our module applies to and obtains better accuracy than existing ConvNets while preserving the model size and computation costs. In this study, all ConvNets models outperform the baseline techniques in all experiments, except in subject-independent experiments on the DEAP dataset. The number of parameters verifies that the learned features are useful for classification. It effectively estimates missing data and maintains good accuracy even when a large proportion of the data is missing. With significantly higher accuracy performance, our MultiT-S ConvNet has approximately six times and three times fewer trainable parameters than Deep ConvNet and Shallow ConvNet, respectively. Despite having fewer parameters than our proposed model, EEGNet performance is unstable in terms of the average accuracy and standard deviation of the SEED and DEAP datasets. Moreover, the additional variations of features extracted by multi-kernel reduce overfitting or vanishing gradient problems while training a model.

## 6. Conclusions

In conclusion, a well-designed end-to-end convolution network is a promising feature extraction and classification tool in EEG-based emotion analysis. We proposed multi-kernel filtering to increase the variation of temporal representations and further recalibrate both temporal and spatial features using the lightweight gating mechanism. The results show that our MultiT-S ConvNet outperforms the traditional and existing models. However, there are some limitations in the current study that could be addressed in future research. This study focused on discrete emotion recognition with the type of movie stimuli. In the future, the applicability of the MultiT-S network will be explored in brain disease recognition, such as epilepsy and Alzheimer’s disease. In addition, this proposed model could be further developed to allow detecting and analyzing dynamic changes in EEG signals over time. Ultimately, our module could be added to any model of EEG-based convolution networks and its ability could improve the learning effect of other existing models when there is a limited dataset.

## Figures and Tables

**Table 1:** The accuracy results of subject-dependent and subject-independent classification on SEED and DEAP datasets. The bottom row indicates our proposed MultiT-S ConvNet results. Bold indicates the highest accuracy.

	SEED-2 Classes		SEED-3 Classes		DEAP-Valence		DEAP-Arousal	
Models	Subj. Dep.	Subj. Indep.	Subj. Dep.	Subj. Indep.	Subj. Dep.	Subj. Indep.	Subj. Dep.	Subj. Indep.
Chance Level	50.0 ± 0.0	50.0 ± 0.0	33.3 ± 0.0	33.3 ± 0.0	56.6 ± 9.2	**56.6 ± 9.2**	58.9 ± 15.5	**58.9 ± 15.5**
KNN	70.5 ± 12.3	72.0 ± 7.8	52.5 ± 12.7	43.7 ± 8.0	55.9 ± 6.9	52.9 ± 5.2	61.6 ± 11.0	53.0 ± 10.7
RF	87.0 ± 7.7	73.4 ± 8.5	71.7 ± 9.5	52.7 ± 6.9	55.6 ± 7.3	51.1 ± 3.6	61.8 ± 10.6	50.0 ± 7.5
FCN	77.4 ± 11.9	66.1 ± 10.1	56.5 ± 13.6	46.4 ± 8.4	54.8 ± 6.4	53.9 ± 6.6	60.8 ± 10.9	50.6 ± 13.2
Deep ConvNet	94.8 ± 3.4	72.0 ± 7.8	85.0 ± 4.5	51.1 ± 7.4	76.6 ± 3.4	47.5 ± 7.0	78.6 ± 4.6	51.8 ± 13.0
Shallow ConvNet	91.2 ± 7.4	74.0 ± 8.5	82.1 ± 7.7	54.4 ± 7.4	76.0 ± 6.9	49.1 ± 7.7	78.9 ± 6.7	51.0 ± 14.7
EEGNet	89.1 ± 3.6	70.1 ± 9.5	76.3 ± 15.1	47.8 ± 9.7	**79.2 ± 6.3**	49.3 ± 7.2	82.1 ± 6.9	51.4 ± 10.3
MultiT-S ConvNet	**95.2 ± 3.2**	**75.1 ± 8.4**	**86.0 ± 5.3**	**54.6 ± 6.8**	77.6 ± 5.8	52.6 ± 8.8	**82.2 ± 6.5**	51.5 ± 10.4

**Table 2:** The number of filters, trainable parameters, and accuracies before and after applying multi-kernel convolution on the SEED dataset.

	No. of Temp. Filter	No. of Spat. Filter	No. of Temp-Spat Filter	Trainable Parameters	Acc.(%)
Deep ConvNet	24	24	48 + 96 + 192 = 336	182,497	72.04 *
Deep ConvNet (+)	12 × 4 = 48	12	24 + 48 + 96 = 168	73,777	73.96 *
Shallow ConvNet	40	40		101,281	73.96
Shallow ConvNet (+)	20 × 4 = 80	20		101,721	73.19
EEGNet	32	64	64	13,537	70.15
EEGNet (+)	16 × 4 = 64	32	32	16,177	74.00 **
MultiT-S ConvNet	24 × 4 = 96	64		30,313	75.13

Note: (+) indicates reforming of temporal filters and halving of spatial filters. Stars indicate paired *t*-tests compared to the corresponding model. (** is *p* < 0.01 and * is *p* < 0.05).

**Table 3:** The comparison of model performance between the proposed MultiT-S ConvNet and existing ConvNets. The accuracy (%) is the average from all experiments in this study. It calculates differences in the accuracy between MultiT-S ConvNet and other models, and the positive mark denotes a higher accuracy of the MultiT-S ConvNet. The minimum frequency (Hz) covered by each model is displayed. The bottom row computes the number of trainable parameters in dependence on the sampling rate of the SEED dataset. The negative and positive marks denote a decrease and increase of trainable parameters required by the MultiT-S ConvNet, compared with indicated models.

	MultiT-S ConvNet	vs. Deep ConvNet	vs. Shallow ConvNet	vs. EEGNet
Accuracy (%)	71.9	+2.2	+2.3	+3.7
Minimum freq. covered	5 Hz	40 Hz	17 Hz	2 Hz
Number of Trainable Parameters	30,313	−152,184	−43,464	+14,136

## Data Availability

The code used in this study is available upon request from the corresponding author.

## References

[1] Al-Nafjan A.N., Hosny M.I., Al-Ohali Y., Al-Wabil A. (2017). Review and Classification of Emotion Recognition Based on EEG Brain-Computer Interface System Research: A Systematic Review. Appl. Sci..

[2] Millan J.d.R., Rupp R., Müller-Putz G., Murray-Smith R., Giugliemma C., Tangermann M., Vidaurre C., Cincotti F., Kübler A., Leeb R. (2010). Combining Brain–Computer Interfaces and Assistive Technologies: State-of-the-Art and Challenges. Front. Neurosci..

[3] Jamil N., Belkacem A.N., Ouhbi S., Lakas A. (2021). Noninvasive Electroencephalography Equipment for Assistive, Adaptive, and Rehabilitative Brain–Computer Interfaces: A Systematic Literature Review. Sensors.

[4] Tangermann M.W., Krauledat M., Grzeska K., Sagebaum M., Vidaurre C., Blankertz B., Müller K.R. (2008). Playing Pinball with Non-Invasive BCI. Proceedings of the 21st International Conference on Neural Information Processing Systems.

[5] Singh A.K., Wang Y.K., King J.T., Lin C.T. (2020). Extended Interaction With a BCI Video Game Changes Resting-State Brain Activity. IEEE Trans. Cogn. Dev. Syst..

[6] Rached T.S., Perkusich A., Fazel-Rezai R. (2013). Emotion Recognition Based on Brain-Computer Interface Systems. Brain-Computer Interface Systems.

[7] Torres E.P., Torres E.A., Hernández-Álvarez M., Yoo S.G. (2020). EEG-Based BCI Emotion Recognition: A Survey. Sensors.

[8] Acharya U.R., Molinari F., Subbhuraam V.S., Chattopadhyay S., Kh N., Suri J. (2012). Automated diagnosis of epileptic EEG using entropies. Biomed. Signal Process. Control.

[9] Asadzadeh S., Yousefi Rezaii T., Beheshti S., Delpak A., Meshgini S. (2020). A systematic review of EEG source localization techniques and their applications on diagnosis of brain abnormalities. J. Neurosci. Methods.

[10] van Vliet M., Robben A., Chumerin N., Manyakov N.V., Combaz A., Van Hulle M.M. Designing a brain-computer interface controlled video-game using consumer grade EEG hardware. Proceedings of the 2012 ISSNIP Biosignals and Biorobotics Conference: Biosignals and Robotics for Better and Safer Living (BRC).

[11] Klimesch W. (1999). EEG alpha and theta oscillations reflect cognitive and memory performance: A review and analysis. Brain Res. Rev..

[12] Schmidt L.A., Trainor L.J. (2001). Frontal brain electrical activity (EEG) distinguishes valence and intensity of musical emotions. Cogn. Emot..

[13] Huang D., Guan C., Ang K.K., Zhang H., Pan Y. Asymmetric spatial pattern for EEG-based emotion detection. Proceedings of the International Joint Conference on Neural Networks (IJCNN).

[14] Jatupaiboon N., Pan-ngum S., Israsena P. (2013). Emotion classification using minimal EEG channels and frequency bands. Proceedings of the 10th international Joint Conference on Computer Science and Software Engineering (JCSSE).

[15] Schirrmeister R.T., Springenberg J.T., Fiederer L.D.J., Glasstetter M., Eggensperger K., Tangermann M., Hutter F., Burgard W., Ball T. (2017). Deep learning with convolutional neural networks for EEG decoding and visualization. Hum. Brain Mapp..

[16] Lawhern V.J., Solon A.J., Waytowich N.R., Gordon S.M., Hung C.P., Lance B.J. (2018). EEGNet: A compact convolutional neural network for EEG-based brain–computer interfaces. J. Neural Eng..

[17] Jenke R., Peer A., Buss M. (2014). Feature Extraction and Selection for Emotion Recognition from EEG. IEEE Trans. Affect. Comput..

[18] Chanel G., Kierkels J.J., Soleymani M., Pun T. (2009). Short-term emotion assessment in a recall paradigm. Int. J.-Hum.-Comput. Stud..

[19] Bos D.O. (2006). EEG-based emotion recognition. Influ. Vis. Audit. Stimuli.

[20] Ramoser H., Muller-Gerking J., Pfurtscheller G. (2000). Optimal spatial filtering of single trial EEG during imagined hand movement. IEEE Trans. Rehabil. Eng..

[21] Ang K.K., Chin Z.Y., Wang C., Guan C., Zhang H. (2012). Filter Bank Common Spatial Pattern Algorithm on BCI Competition IV Datasets 2a and 2b. Front. Neurosci..

[22] Zheng W.L., Lu B.L. (2015). Investigating Critical Frequency Bands and Channels for EEG-based Emotion Recognition with Deep Neural Networks. IEEE Trans. Auton. Ment. Dev..

[23] Li M., Lu B.L. Emotion classification based on gamma-band EEG. Proceedings of the 2009 Annual International Conference of the IEEE Engineering in Medicine and Biology Society.

[24] Candra H., Yuwono M., Chai R., Handojoseno A., Elamvazuthi I., Nguyen H.T., Su S. Investigation of window size in classification of EEG-emotion signal with wavelet entropy and support vector machine. Proceedings of the 2015 37th Annual International Conference of the IEEE Engineering in Medicine and Biology Society (EMBC).

[25] Shu L., Xie J., Yang M., Li Z., Li Z., Liao D., Xu X., Yang X. (2018). A review of emotion recognition using physiological signals. Sensors.

[26] Hu J., Shen L., Sun G. (2017). Squeeze-and-Excitation Networks. IEEE Trans. Pattern Anal. Mach. Intell..

[27] Koelstra S., Mühl C., Soleymani M., Lee J.S., Yazdani A., Ebrahimi T., Pun T., Nijholt A., Patras I. (2011). DEAP: A Database for Emotion Analysis Using Physiological Signals. IEEE Trans. Affect. Comput..

[28] Abadi M., Agarwal A., Barham P., Brevdo E., Chen Z., Citro C., Corrado G.S., Davis A., Dean J., Devin M. (2015). TensorFlow: Large-Scale Machine Learning on Heterogeneous Systems. tensorflow.org.

[29] (2015). Keras. https://github.com/fchollet/keras.

[30] Emsawas T., Kimura T., Fukui K.i., Numao M. Comparative Study of Wet and Dry Systems on EEG-Based Cognitive Tasks. Proceedings of the International Conference on Brain Informatics.

[31] Hagad J.L., Kimura T., Fukui K.i., Numao M. (2021). Learning subject-generalized topographical EEG embeddings using deep variational autoencoders and domain-adversarial regularization. Sensors.

